# Special Patterns of Dynamic Brain Networks Discriminate Between Face and Non-face Processing: A Single-Trial EEG Study

**DOI:** 10.3389/fnins.2021.652920

**Published:** 2021-06-09

**Authors:** Zhongliang Yin, Yue Wang, Minghao Dong, Shenghan Ren, Haihong Hu, Kuiying Yin, Jimin Liang

**Affiliations:** ^1^School of Electronic Engineering, Xidian University, Xi'an, China; ^2^Engineering Research Center of Molecular and Neuro Imaging of Ministry of Education, School of Life Science and Technology, Xidian University, Xi'an, China; ^3^Nanjing Research Institute of Electronics Technology, Nanjing, China

**Keywords:** face processing, dynamic brain network, minimum spanning tree, electroencephalography, classification

## Abstract

Face processing is a spatiotemporal dynamic process involving widely distributed and closely connected brain regions. Although previous studies have examined the topological differences in brain networks between face and non-face processing, the time-varying patterns at different processing stages have not been fully characterized. In this study, dynamic brain networks were used to explore the mechanism of face processing in human brain. We constructed a set of brain networks based on consecutive short EEG segments recorded during face and non-face (ketch) processing respectively, and analyzed the topological characteristic of these brain networks by graph theory. We found that the topological differences of the backbone of original brain networks (the minimum spanning tree, MST) between face and ketch processing changed dynamically. Specifically, during face processing, the MST was more line-like over alpha band in 0–100 ms time window after stimuli onset, and more star-like over theta and alpha bands in 100–200 and 200–300 ms time windows. The results indicated that the brain network was more efficient for information transfer and exchange during face processing compared with non-face processing. In the MST, the nodes with significant differences of betweenness centrality and degree were mainly located in the left frontal area and ventral visual pathway, which were involved in the face-related regions. In addition, the special MST patterns can discriminate between face and ketch processing by an accuracy of 93.39%. Our results suggested that special MST structures of dynamic brain networks reflected the potential mechanism of face processing in human brain.

## 1. Introduction

Face processing is a well-developed human capability, and thanks to it, we can recognize faces more quickly and accurately than non-face objects in complex environments. In the past decades, the distinct processing mechanism of faces in the brain has been extensively explored by neuroscientists and psychologists (Bentin et al., 1996; Ishai et al., 2005; Yang et al., 2015; Uono et al., 2017; Fan et al., 2020; Muukkonen et al., 2020; Wang et al., 2020; Yin et al., 2020). It is well-accepted that face processing is a spatiotemporally dynamic process involving widely distributed and closely connected brain regions (Ishai et al., 2005; Uono et al., 2017; Muukkonen et al., 2020). From the perspective of temporal dimension, the event related potential (ERP) studies show that face processing is distinctive from non-face processing in human brain in different time windows (Bentin et al., 1996; Yang et al., 2015). Specifically, the amplitude of P1 occurring around 80–100 ms after stimuli onset is larger for faces than for non-face objects; the amplitude of N1 increases and the latency of N1 is about 30 ms longer for faces (arising around 160–190 ms) than for non-face objects (arising around 130–160 ms). From the spatial dimension, researches with high spatial resolution (such as fMRI) propose that the neural activities in and between the face-related brain regions are distinctive compared with non-face processing (Ishai et al., 2005; Axelrod and Yovel, 2013; Renzi et al., 2013; Duchaine and Yovel, 2015; Muukkonen et al., 2020), demonstrating that these regions and their connections construct a unique topology for face processing. However, few studies have investigated the time-varying patterns of the topology at different processing stages to fully characterize the mechanism of face processing.

The human brain is a complex network with highly connected and widely distributed regions (Meunier et al., 2010; van den Heuvel and Sporns, 2011; Bullmore and Sporns, 2012). Analyses based on graph theoretical approaches suggest that human brain networks are organized according to an efficient topology that integrates brain regions with similar function into groups (called communities) and maintains short path lengths between regions, i.e., the small-world organization (Sporns and Honey, 2006; Bullmore and Sporns, 2012). The efficient topology of brain networks has been considered to play a key role in cognitive processing, and different topologies are associated with specific stages of the cognitive process (Zhang et al., 2019; Allegra et al., 2020; Delgado Reyes et al., 2020; Finc et al., 2020; Si et al., 2020).

Topological organization analysis of brain networks characterizes the integration and segregation of information between distributed brain regions, whereas dynamic brain networks investigate changes in the topological organization of a set of brain networks which are typically constructed from brain activities recorded in sequential time windows. When employed to study human cognitive processes, dynamic brain networks enable the integration of multiple dimensions of the information about neural activities in the brain (Bola and Sabel, 2015; Hassan et al., 2015; Rizkallah et al., 2018). In this study, we investigated the differences in brain networks corresponding to face processing and non-face processing in different time windows. The goal is to understand how the differences change in different processing stages, thus enabling us to reveal the underlying mechanism of face processing from a spatiotemporal perspective.

When comparing the topologies of graph networks, the conventional operations suffer from some methodological problems (van Wijk et al., 2010; Fornito et al., 2013; Tewarie et al., 2015). Specifically, for binary network analysis, the networks being compared are obtained by binarizing the original weighted networks using a fixed threshold, which may change the average degree of the networks differently. As the graph measures are influenced by the average degree of network, the comparison may lead to biased results. Although weighted network analysis can alleviate the bias caused by the thresholding operation, the difference in the averaged weights will influence the graph measures of weighted networks. Recently, the minimum spanning tree (MST) has been adopted to tackle the aforementioned issues (Stam et al., 2014; Tewarie et al., 2015; van Dellen et al., 2018). The MST is a sub-network without loops and is the backbone of the original network. It has been used to explore the mechanism of mental illnesses and cognitive functions (Fraschini et al., 2016; Tóth et al., 2017; Utianski et al., 2017; Cao et al., 2020; Das and Puthankattil, 2020). Moreover, various measures of MST were used as features to distinguish different cognitive states or different groups via machine learning methods (Cui et al., 2018; Guo et al., 2018; Mehraram et al., 2019; Saba et al., 2019).

In this study, we made an exploratory research on the mechanism of face processing by dynamic brain networks based on electroencephalography (EEG) recordings. Firstly, the EEG data recorded during face and non-face (ketch) processing were filtered into 5 classical frequency bands, including delta (0.5–4 Hz), theta (4–8 Hz), alpha (8–12 Hz), beta (13–30 Hz), and gamma (31–46 Hz) bands. Secondly, trials were extracted and each trial was divided into 5 time windows with the length of 100 ms. Thirdly, MSTs of the brain networks corresponding to face and ketch processing in each time window over each frequency band were constructed. Fourthly, the MST topologies of face and ketch processing were compared and the MST measures with significant differences were selected as features. Finally, the discriminability of the selected MST measures was validated by a classification model using the support vector machine (SVM).

## 2. Materials and Methods

### 2.1. Participants

Twenty-eight volunteers (15 male, 13 female; age = 27.41 ± 5.47, mean ± standard deviation) were recruited from the Xidian Community. They were all right-handed, reported normal or corrected to normal visual acuity, and did not have any history of psychiatric or neurological disorders. All the volunteers provided written informed consents and received monetary payment for the participation. The experimental procedures complied with Helsinki Declaration of 1975 which was revised in 2000.

### 2.2. Experimental Design

**Figure 1:**
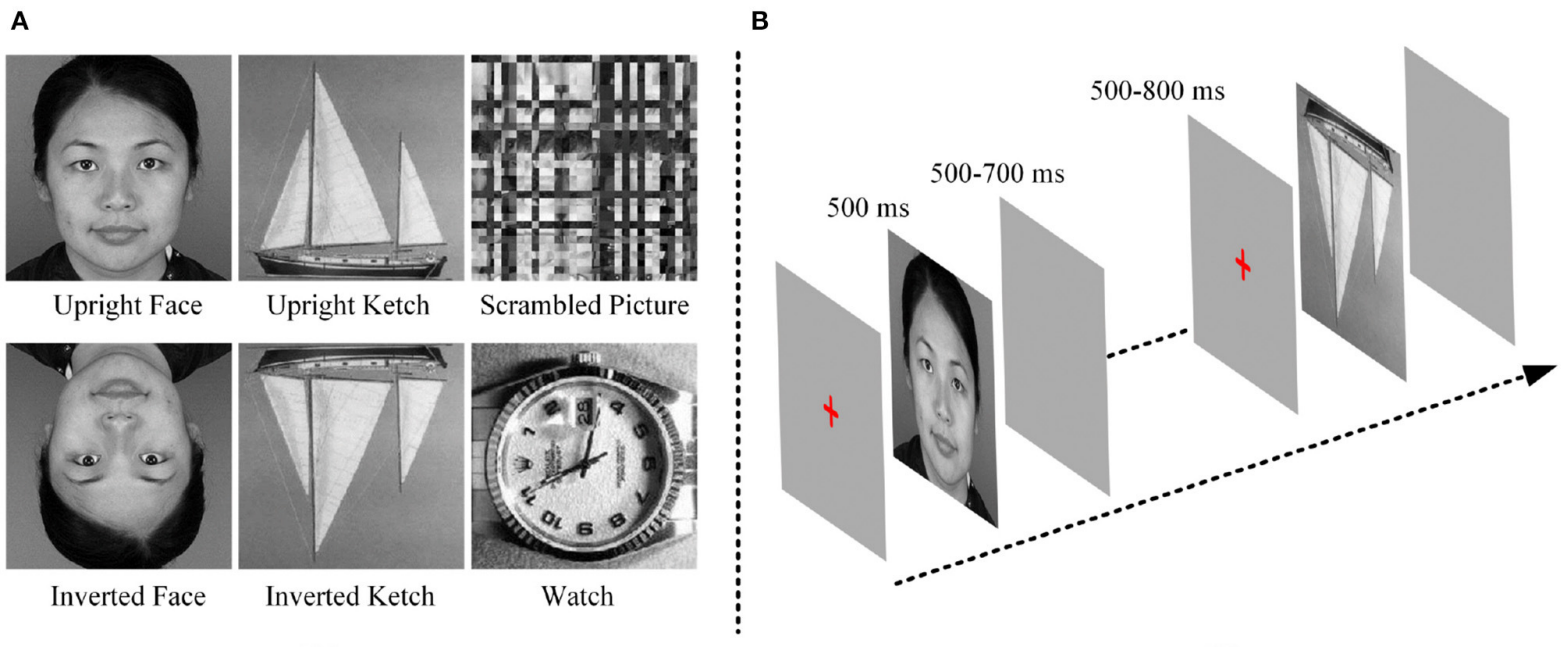
**(A)** Examples of each kind of stimuli. **(B)** Procedure of experiment.

The stimuli were three categories of pictures, including 48 faces (24 males and 24 females), 48 ketches and 48 watches. Face and ketch pictures were presented in two conditions: upright and inverted. In addition, 48 scrambled pictures were included as the baseline stimuli. The scrambled pictures were obtained from face pictures by two steps. First, each picture was divided into small blocks, and then, the small blocks were rearranged until the identity of the scrambled picture could not be discerned. The face pictures were collected from public-accessed websites, the ketch and watch pictures were chosen from the Caltech256 image set (Griffin et al., 2006). To eliminate the influence of physical properties of stimuli, grayscale pictures were used, and all of them were cropped into 300 × 300 pixels. Pixel values of pictures were normalized to make them have similar luminance levels. Moreover, the object in each picture occupied as least 75% space of the picture. Figure 1 shows examples of the stimuli.

The experiment was conducted in an electromagnetically shielded and semi-dark room. The introduction and stimuli were presented on an LCD screen (resolution: 1980 × 1080 pixels, refresh rate: 60 Hz). The participants were positioned at a viewing distance of approximately 80 cm and seated in a comfortable armchair with their eyes fixating on the center of the screen. The stimuli were presented using E-Prime 2.0 presentation software running under Windows 7 with an Nvidia GeForce GTX 750 graphics card.

During the experiment, before a stimulus was presented, a red cross was displayed in the center of the screen for 500–800 ms. Each stimulus was presented for 500 ms and followed by an empty screen for 500–700 ms. The red-cross, stimulus and empty screen constituted an experimental trial the duration of which was approximately 1,750 ms. All the stimuli were divided into 3 blocks. Each block included 96 different stimuli (16 upright faces, 16 inverted faces, 16 upright ketches, 16 inverted ketches, 16 watches and 16 scrambled pictures), and lasted approximately 2.8 min. Each block was repeated six times, and the pictures were shuffled in each block. There were 18 blocks in this experiment, with 288 trials for each type of pictures, and the total time was about 70 min. The participants were requested to observe the stimuli carefully and make keyboard responses during the empty screen after they saw the watches. During the experiment, after one block was finished, the experiment was paused. Then, the subject could pressed the “space key” to go on or had a short break when he felt tired. The block design avoided the subjects' fatigue. The experimental procedure was similar to the ones used in previous studies (Bentin et al., 1996; Uono et al., 2017), in order to keep them consistent, the inverted faces and ketches were included. The goal of our study was to investigate the potential mechanism of face processing by compared with non-face processing, so the EEG data of inverted faces and ketches was not analyzed in this study. Figure 1 shows the experimental procedure.

### 2.3. EEG Recording and Preprocessing

When the participants undertook the task, EEG data were recorded using a 64-channel amplifier provided by Brain Products Company (ActiCHamp system). All the channels, including a reference channel (channel Iz) and 63 EEG channels, were deployed over the head according to the international 10-10 system (Robert and Peter, 2001; Jurcak et al., 2007). The sampling rate was set to 1,000 Hz, the contact impedance of each channel was kept below 10kΩ, the pass-band filter with 0.5–100 Hz and notch filter with 50 Hz were employed when EEG data were recorded.

The EEG data were preprocessed off-line in the following steps. Firstly, EEG data were re-referenced to the common average reference and inspected by visual observation using EEGLAB (Delorme and Makeig, 2004) to remove epochs containing artifacts such as slow drift. Secondly, the electrooculogram (EOG), electromyography (EMG) and other non-cognitive related artifacts were removed by independent component analysis (ICA). Thirdly, five narrowband filters were used to obtain 5 classical frequency-band EEG data [delta (0.5–4 Hz), theta (4–8 Hz), alpha (8–12 Hz), beta (13–30 Hz), gamma (31–46 Hz)]. In this study, the delta, theta, alpha, and beta frequency bands were similar with other EEG studies on face processing (Sakihara et al., 2012; Yu et al., 2016; Tóth et al., 2017). The gamma bands were defined according to face studies (Gao et al., 2013; Uono et al., 2017; Frauscher et al., 2018). Finally, trials were extracted according to the type of stimuli, ranging from −200 ms before to 800 ms after the stimulus onset. Each trial was baseline-corrected by subtracting the mean amplitude over the epoch between −200 and 0 ms.

In this study, the total number of the trials of all the subjects per category was 8064 (28 × 48 × 6). After preprocessed, noisy trials were removed. Then, we chose nearly equal number of trials corresponding to faces, ketches, and scrambled pictures for each subject for further analysis. The number of the trials of each subject per category was about 274. Finally, the total number of trials was 7,665 per category. The dataset was randomly divided into two subsets, where the first subset contained 70% of trials (5,366 trials per category) and the second contained the remaining 30% of trials (2,299 trials per category). The first subset was used to investigate the distinctive processing of face compared with non-face (ketch) through dynamic brain network analysis. The second one was used to test the discriminatory ability of brain network features in face and non-face processing classification.

### 2.4. Data Analysis

**Figure 2:**
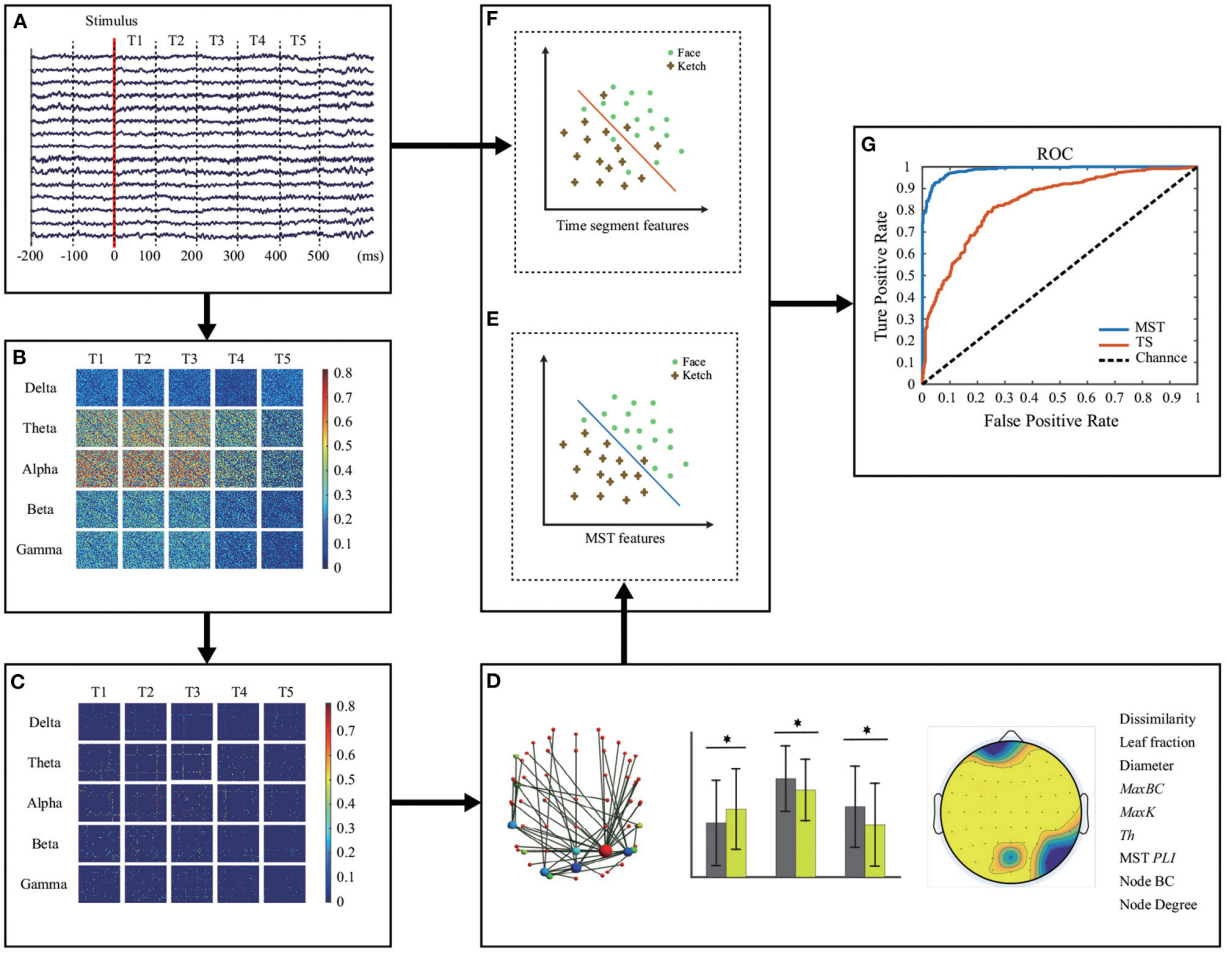
Methodological workflow. **(A)** EEG recording and pre-processing. Five frequency bands of EEG data were calculated and divided equally into 5 time windows. **(B)** Original brain network construction for each time window and each frequency band. **(C)** MST construction. **(D)** MST representation and analysis. Measures with significant differences between face and ketch processing were concatenated as features for classification. **(E,F)** Classification of face and ketch processing using MST features and EEG time segments, respectively. **(G)** Evaluation of classification performance.

Figure 2 shows the methodological workflow for data analysis in this study. The data processing and analysis were performed in MATLAB 2015b including EEGLAB (version 13_6_5b, http://sccn.ucsd.edu/eeglab/).

#### 2.4.1. Time Window Division

For each trial, the data segment from 0 to 500 ms after stimulus onset was selected for analysis. Each data segment was divided equally into 5 time windows (0–100, 101–200, 201–300, 301–400, 401–500 ms), denoted as T1 to T5, respectively. In each time window, a brain network was constructed over each frequency band separately.

In this study, the length of 100 ms was used for two reasons. First, we found that longer data segment may lead to severe averaging effects that reduced the temporal resolution, while too short data segments caused excessive computational load. Second, the P1-N170-P2 effect of face processing indicates that the unique processing of face may occur in 0–100, 100–200, and 200–300 ms sequentially (Gu et al., 2010; Yang et al., 2015).

#### 2.4.2. Functional Connectivity

The phase lag index (*PLI*) (Stam et al., 2007) was used to estimate the strength of correlations between EEG channels that, in neuroscience research, is referred to as functional connectivity. The *PLI* evaluates the phase synchronization between EEG signals of two channels. Before calculating *PLI*, the instantaneous phases of an EEG channel data *x*(*t*) should be obtained from the corresponding analytical signal *z*(*t*) which is a complex signal with a real part of *x*(*t*) and an imaginary part of $\tilde{x}\left(t\right)$:

(1) $$\begin{array}{r} z\left(t\right)=x\left(t\right)+i\tilde{x}\left(t\right)=A\left(t\right)e^{i\phi \left(t\right)}, \end{array}$$

where $\tilde{x}\left(t\right)$ is the Hilbert transform of *x*(*t*). The instantaneous amplitude *A*(*t*) and phase ϕ(*t*) of *x*(*t*) can be calculated as

(2) $$\begin{array}{r} A\left(t\right)=\sqrt{{\left[\tilde{x}\left(t\right)\right]}^{2}+{\left[x\left(t\right)\right]}^{2}}, \end{array}$$

(3) $$\begin{array}{r} \phi \left(t\right)=arctan\frac{\tilde{x}\left(t\right)}{x\left(t\right)}. \end{array}$$

The *PLI* between EEG signals of two channels is computed as

4() $$PLI=\left|\frac{1}{N}\sum_{k=1}^{N}sign\left\{\sin\left[\Delta \phi (t_{k})\right]\right\}\right|,$$

where Δ*Φ*(*t*_*k*_) is the phase difference between two time series at time instant *k*, *N* is the total number of samples, “*sign*” refers to the Heaviside function, and “*sin*” is the sinusoidal function. As the “*sign*” function is introduced in Equation (4), the zero-lag synchronization is removed, which makes the *PLI* less affected by the volume conduction effect compared with other methods (Stam et al., 2007). The value of *PLI* ranges from 0 to 1. A *PLI* value of 0 indicates either no coupling or coupling with phase difference locking at a value different from 0, such that the functional connectivity will be 0. The larger the *PLI*, the stronger the functional connectivity. More details of the *PLI* computation can be found in Stam and his colleagues' work (Stam et al., 2007).

The functional connectivities of channel pairs constitute the adjacency matrix of a brain network. For each trial, each frequency band and each time window, a brain network was constructed accordingly.

#### 2.4.3. MST Construction and Representation

To investigate the topological organization of the brain networks, the MST of each brain network was constructed and the graph theoretical representations of the MSTs were analyzed. Only a brief description of the MST is given here, the detailed information can be found in previous studies (Stam et al., 2014; Tewarie et al., 2015; van Dellen et al., 2018).

MST is a sub-network of the original network in which all nodes are connected without forming loops and has the minimum total weight of all possible spanning trees (Tewarie et al., 2015). Since the *PLI* values, which can be considered as an inverse distance, were used in this study as the link weights for the original brain network, we retained the maximum link weights to construct the MSTs (formally a maximum spanning tree) using the Kruskal's algorithm (Kruskal, 1956). Specifically, given an original network, the weights of all its links were sorted and the link with the maximum weight was added to the MST. Then, the link with the next largest weight was added, and this process continued until all nodes were connected. If adding a link resulted in the formation of a loop, the link was skipped. At the end of the link addition process, a weighted MST was generated. Finally, the weighed MST was binarized.

Several measures are usually used to characterize the MST, including degree (*K*), leaf fraction (*L*_*f*_), eccentricity (*E*), diameter (*D*), betweenness centrality (*BC*), and tree hierarchy (*Th*). The detailed information of these measures can be found in Boersma et al. (2013), Stam et al. (2014), and Tewarie et al. (2015). In addition to the graph measures, the sum of *PLI* (denoted as MST *PLI*) is also an important measure of MST, which estimates the degree of regional coupling in the MST.

There are two extreme structures of MST—line and star structures, corresponding to regular and random networks, respectively. For an MST with *N* nodes and *N-1* links, the two extreme structures have deterministic measures. For a line structure, the maximum of degree *K* is 2, the number of leaf nodes is 2 and the diameter *D* is *N-1*. For a star structure, the maximum of *K* is *N-1*, the number of leaf nodes is *N-1*, and *D* is 2. An MST is considered as line-like if its measures are closer to that of a line structure than to a star structure, otherwise it is star-like. In general, the structure of a brain network is between a line and a star structure.

When comparing the topologies of two MSTs, the dissimilarity between them should first be estimated. In this study, the dissimilarity is quantified using a measure based on the information theory. For two different MSTs (*MST*_*n*_ and *MST*_*m*_) with the same number of nodes, the dissimilarity measure computes how much information is needed, on average, to explain *MST*_*n*_ given *MST*_*m*_ (Lee et al., 2006). The dissimilarity is defined as

(5) $$S_{n/m}=\frac{1}{N}\sum_{i=1}^{N}log_{10}\;\left|\frac{S_{n(i)}}{S_{m(i)}}\right|,$$

where *S*_*n*(*i*)_ and *S*_*m*(*i*)_ are the sum of distances from a reference node *i* to all its neighbors in *MST*_*n*_ and *MST*_*m*_, respectively. Distances refer to the path length based on the *PLI*. More detailed explanations about the dissimilarity can be found in Lee et al. (2006) and Tewarie et al. (2014).

In this study, the dissimilarity of MSTs between face and ketch processing was evaluated through a reference *MST*_*ref*_. Specifically, a reference *MST*_*ref*_ was firstly constructed from the network obtained by averaging the adjacency matrixes corresponding to all the scrambled pictures. Then, the dissmilarities of MSTs during face (*MST*_*x*_) and ketch processing (*MST*_*y*_) compared with the reference *MST*_*ref*_ (*S*_*x*/*ref*_ and *S*_*y*/*ref*_) were calculated, respectively. Finally, *S*_*x*/*ref*_ and *S*_*y*/*ref*_ was compared to evaluate the dissimilarity of MSTs betweeen face and ketch processing.

#### 2.4.4. Statistical Analysis

Wilcoxon rank sum test was conducted for each measure of the MSTs between face and ketch stimuli in each time window and each frequency band. The dissimilarity between the corresponding MSTs was analyzed firstly. Further analyses were carried out only for the pairs of MSTs demonstrating significant dissimilarity. The measures including *D*, *L*_*f*_, maximum *BC* (*MaxBC*), maximum degree (*MaxK*), *Th*, MST *PLI*, and each node's *BC* and *K* were calculated. Wilcoxon rank sum test was performed for each measure and the *p*-values were corrected by false discovery rate (FDR). The correction took two factors into account - the time window (T1, T2, T3, T4, T5) and the frequency band (delta, theta, alpha, beta, and gamma). Moreover, for the *K* and *BC* of each node, the number of nodes was also considered. In this study, the significant level was set to 0.05.

In this study, Wilcoxon rank sum test with FDR correction was chosen for the statistical analysis for two reasons. Frist, Wilcoxon rank sum test does not require the data to follow a normal distribution. Second, since there were several factors (including time windows and frequency bands) that influenced the analysis results, the multiple comparison correction should be used to correct the statistical results. There are two routine methods of multiple comparison correction, FDR and Bonferroni correction. Bonferroni correction is stricter than FDR correct, but it often rejects not only false positives but also many positive results (Benjamini and Yekutieli, 2001), so we used FDR.

In summary, in this study, the EEG data were firstly filtered into five frequency bands and then each original EEG trial was divided into five time segments. For each time segment, a brain network was constructed based on the adjacency matrix defined by phase lagged index. After the brain networks were constructed, we extracted MSTs of the brain networks and calculated their measures. Then, we compared and analyzed the topology of MSTs corresponding to faces and ketches in each time window over each frequency band across subjects.

### 2.5. Classification Based on SVM

A machine learning approach was used to validate the single-trail discriminatory ability of the MST measures for face and non-face processing classification. After analysis of the first dataset, all the MST measures with significant differences between the two types of stimuli across all time windows and frequency bands were concatenated as features. For the second dataset, we calculated the network features, and a SVM classifier was adopted for classification. The total number of the second dataset was 2299 for face and ketch stimuli respectively. We performed five-fold cross-validation to train SVM classifiers using the LIBSVM library (Chang and Lin, 2011) and reported the results averaged over 5 repetitions. For comparison with the MST measures, we also used the EEG time segments (TS) over occipito-temporal areas as features for SVM classification. Details about the construction of TS samples was presented in the Supplementary Material. The classification performance was quantified using the receiver operating characteristic (ROC) curve, mean accuracy, sensitivity, specificity, and area under the ROC curve (AUC).

## 3. Results

### 3.1. MST Dissimilarity Test

**Figure 3:**
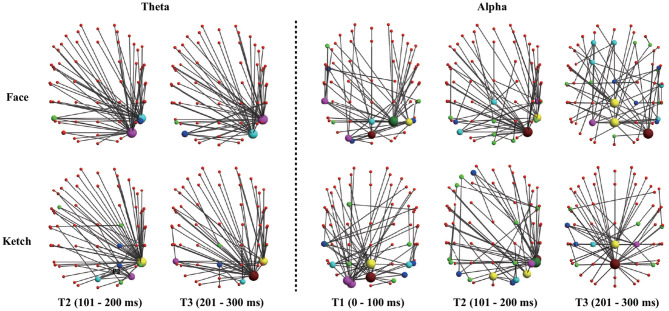
Sketch maps of MSTs of face and ketch processing in axial view (dorsal side). The top part corresponds to anterior part of brain, and left part corresponds to left part of brain.

The results of dissimilarity test demonstrated that there were significant differences in the processing of faces and ketches in certain time windows and frequency bands, as shown in Table 1. Specifically, over theta band, the dissimilarity presented in T2 and T3; over alpha band, the dissimilarity presented in T1, T2, and T3. Figure 3 shows the sketch maps of MSTs of face and ketch processing in different windows over theta and alpha bands. The MSTs were constructed from the adjacent matrices averaged from all trails of each situation, respectively. The balls represent the nodes (channels) and their size is the degree of the node. The red balls are leaf nodes, other colored balls are hub nodes with degree greater than 1. The larger the size of the ball, the greater the node's degree, and the more important the hub node is in the MST. The sketch maps were made by BrainNet Viewer (Xia et al., 2013).

**Table 1:** Results of MST dissimilarity test.

**Stimulus**	**Theta**		**Alpha**		
	**T2**	**T3**	**T1**	**T2**	**T3**
Face/ref	−0.0699 ± 0.0299	−0.0656 ± 0.0307	−0.0728 ± 0.0296	−0.0832 ± 0.0269	−0.0774 ± 0.0290
Ketch/ref	−0.0643 ± 0.0307	−0.0636 ± 0.0310	−0.0762 ± 0.0283	−0.0795 ± 0.0276	−0.0741 ± 0.0289
*p*-value	2.8199e-21	5.0350e-04	3.6358e-09	9.8099e-14	3.9975e-11

*Only results with significant differences are shown. The measure values are in the form of mean ± std*.

### 3.2. MST Measure Analysis

**Figure 4:**
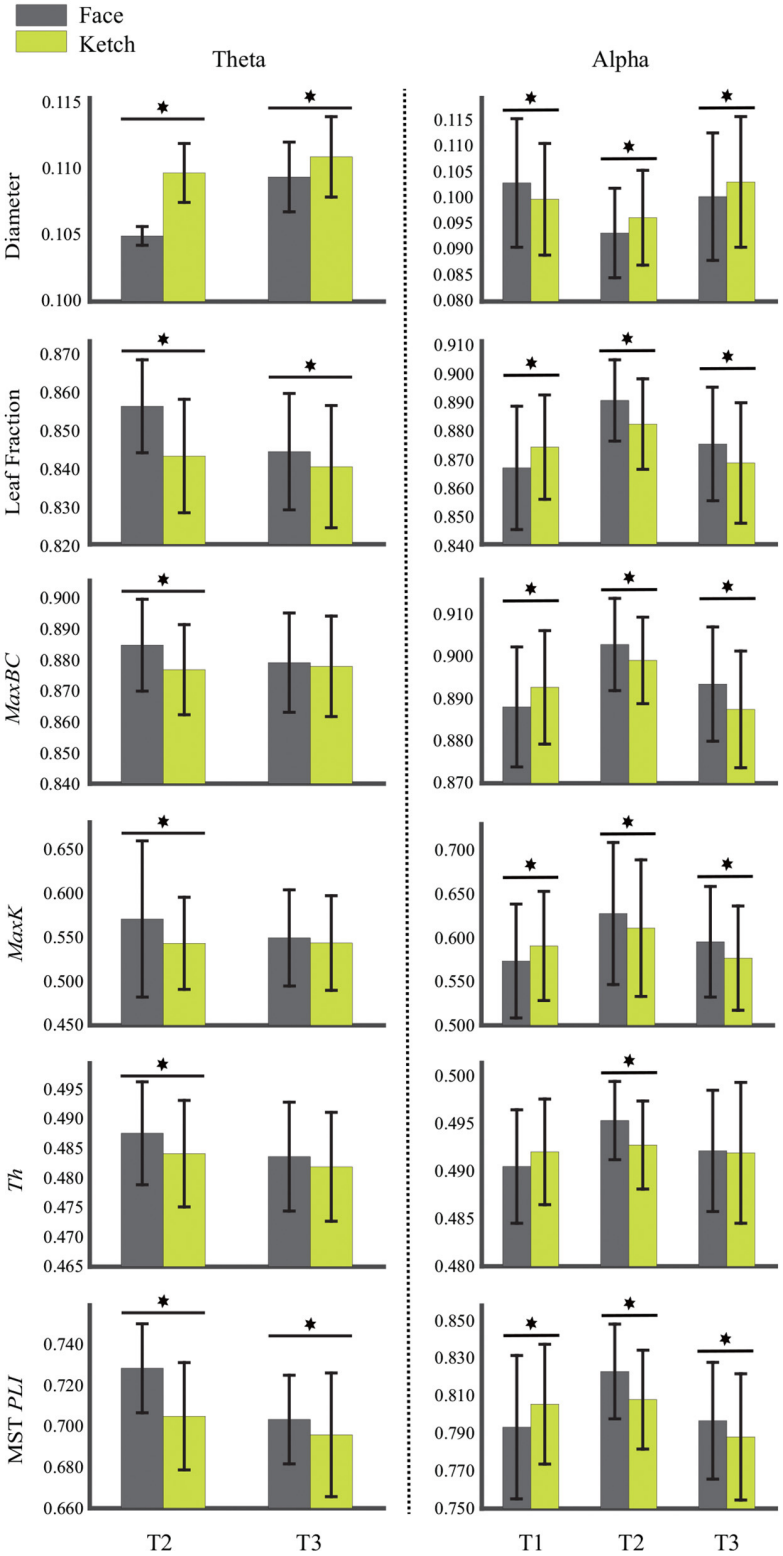
Diameter, leaf fraction, *MaxBC, MaxK, Th*, and MST *PLI* of MSTs of face and ketch processing in different time windows over theta and alpha frequency bands. The asterisks indicate results with significant differences after FDR correction, and bars represent standard error.

Table 2 and Figure 4 present the statistical results of the MST measures of face processing compared with ketch processing. From these, we can draw the following observations.

**Table 2:** Results of MST measure test.

**MST measure**	**Stimulus**	**Theta**		**Alpha**		
		**T2**	**T3**	**T1**	**T2**	**T3**
D	Face	0.1053 ± 0.0308	0.1095 ± 0.0326	0.1028 ± 0.0322	0.0928 ± 0.0284	0.0998 ± 0.0323
	Ketch	0.1104 ± 0.0324	0.1115 ± 0.0331	0.0995 ± 0.0303	0.0964 ± 0.0294	0.1026 ± 0.0321
	*p*-value	2.2327e-16*	0.0012*	2.2082e-09*	2.3559e-11*	5.8504e-08*
*L*_*f*_	Face	0.8566 ± 0.0730	0.8450 ± 0.0760	0.8657 ± 0.0711	0.8907 ± 0.0638	0.8757 ± 0.0697
	Ketch	0.8421 ± 0.0758	0.8397 ± 0.0769	0.8736 ± 0.0676	0.8812 ± 0.0660	0.86831 ± 0.0700
	*p*-value	1.0381e-24*	0.0002*	1.7478e-08*	7.2764e-16*	3.0733e-10*
*MaxBC*	Face	0.8847 ± 0.0853	0.8788 ± 0.0865	0.8887 ± 0.0847	0.9043 ± 0.0809	0.8942 ± 0.0844
	Ketch	0.8757 ± 0.0851	0.8768 ± 0.0870	0.8925 ± 0.0842	0.8984 ± 0.0817	0.8886 ± 0.0838
	*p*-value	2.5066e-10*	0.2077	0.0060*	5.2097e-06*	7.0164e-06*
*MaxK*	Face	0.5506 ± 0.1780	0.5313 ± 0.1778	0.5610 ± 0.1784	0.6151 ± 0.1747	0.5843 ± 0.1790
	Ketch	0.5222 ± 0.1743	0.52366 ± 0.1767	0.5765 ± 0.1765	0.5946 ± 0.1738	0.5650 ± 0.1740
	*p*-value	5.4360e-16*	0.0285	7.5457e-06*	6.9950e-10*	8.8900e-09*
*Th*	Face	0.4870 ± 0.0489	0.4836 ± 0.0494	0.4898 ± 0.0462	0.4951 ± 0.0440	0.4925 ± 0.0469
	Ketch	0.4837 ± 0.0495	0.4817 ± 0.0496	0.4923 ± 0.0457	0.4931 ± 0.0450	0.4914 ± 0.0472
	*p*-value	0.0001*	0.0335	0.0306	0.0046*	0.2048
MST *PLI*	Face	0.7268 ± 0.1117	0.7033 ± 0.1168	0.7915 ± 0.0981	0.8231 ± 0.0849	0.7961 ± 0.0909
	Ketch	0.7024 ± 0.1158	0.6942 ± 0.1193	0.8035 ± 0.0908	0.8057 ± 0.0862	0.7875 ± 0.0921
	*p*-value	2.3108e-32*	2.7399e-06*	1.0424e-06*	3.2710e-23*	0.0004*

*The asterisks indicate results with significant differences after FDR correction. The measure values are in the form of mean ± std*.

Over theta band, in T2, the face processing had a significantly shorter MST diameter compared with ketch processing, whereas the other measures (including *L*_*f*_, *MaxBC, MaxK, Th*, and MST *PLI*) were significantly larger. In T3, the MST diameter of face processing was also significantly shorter than that of ketch processing, but unlike in T2, only the leaf fraction and MST *PLI* were significantly larger than that of ketch processing. These results indicated that the structures of MSTs were more star-like during face processing compared with during ketch processing in T2 and T3 over theta band.

Over alpha band, in T1, the MST diameter was significantly longer during face processing compared with ketch processing, while the other measures (except for *Th*) were significantly smaller. These results indicated that the structure of MST was more line-like during face processing. In contrast, in T2 and T3, the MST diameter was significantly shorter and the other measures were significantly larger (except for *Th* in T3). These results indicated that the MST structures were more star-like during face processing in T2 and T3 over alpha band.

**Figure 5:**
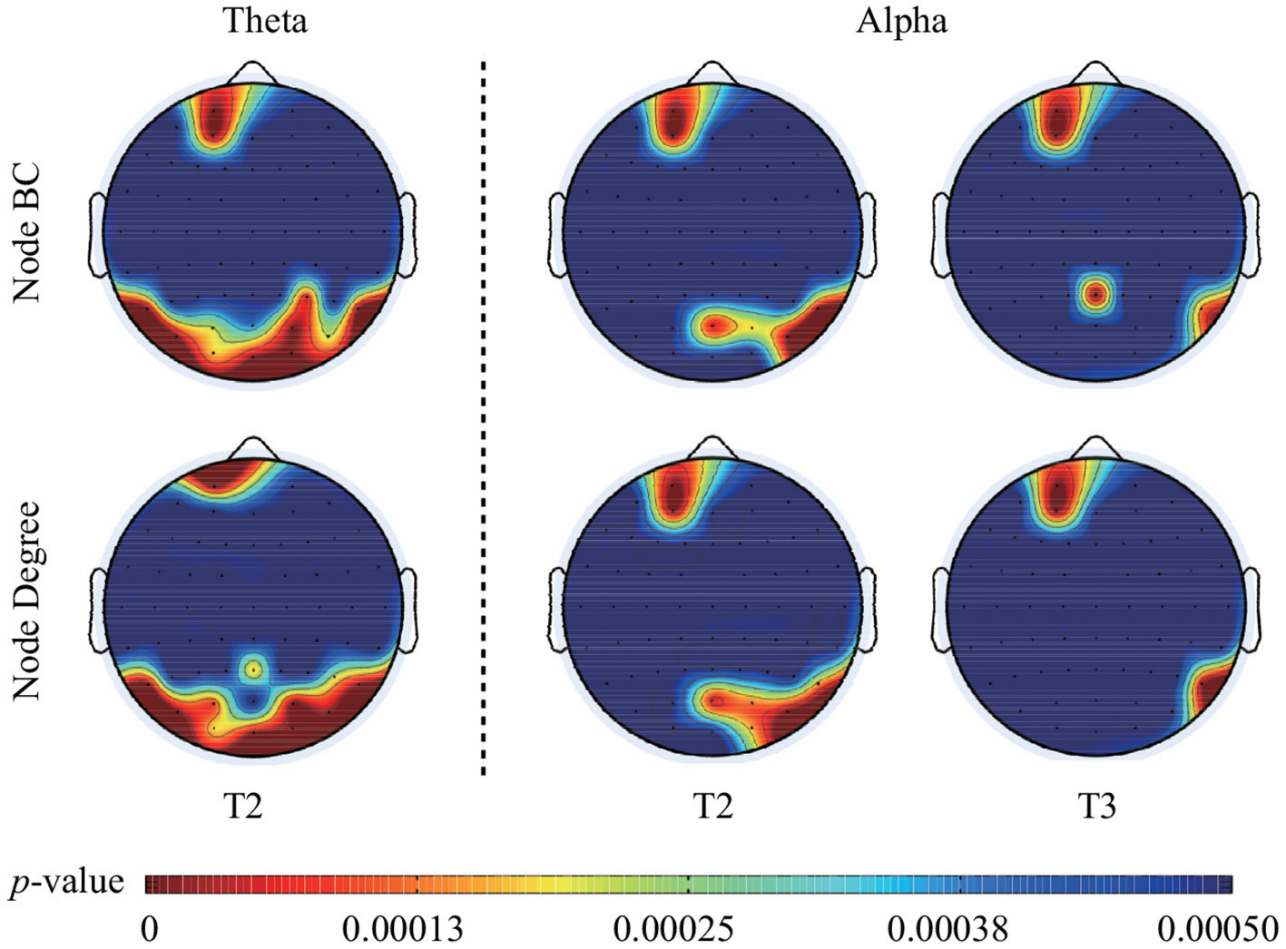
Topographic mapping of the *p*-values of *BC* and degree of each node in T2 over theta band, in T2 and T3 over alpha band. After FDR correction, the critical *p*-value of *BC* value and node degree was 0.00037 in T2 over theta band, 0.00018 in T2 and 0.00014 in T3 over alpha band.

The statistical analysis of node *BC* presented that some nodes had significantly larger *BC* values during face processing than that during ketch processing. Specifically, over theta band and in T2, these nodes were mainly located in the left frontal area, and bilateral ventral visual pathway of the brain (including Fp1, AF3, TP9, P7, PO7, PO3, O1, POz, Oz, O2, P4, P8, TP10, PO4, and PO8). Over alpha band, in T2, these nodes appeared in the left frontal region (Fp1 and AF3), and right occipito-temporal region (POz, PO4, PO8, P8, and TP10); in T3, these nodes mainly appeared in the left frontal region (Fp1 and AF3), and right parietal-temporal region (Pz, P8, and TP10). The results of node degree were similar to the results of *BC*. Several nodes presented significant larger degree during face processing than that during ketch processing. Over theta band, in T2, the nodes were Fp1, PO3, PO7, P7, O1, Pz, Oz, O2, PO4, PO8, P6, P8, and TP10. Over alpha band, in T2, the nodes were Fp1, AF3, POz, O2, PO4, PO8, P8, and TP10; in T3, the nodes were Fp1, AF3, P8, and TP10. Figure 5 shows the topographic mapping of the *p*-values of node *BC* and degree, respectively.

### 3.3. Classification

**Figure 6:**
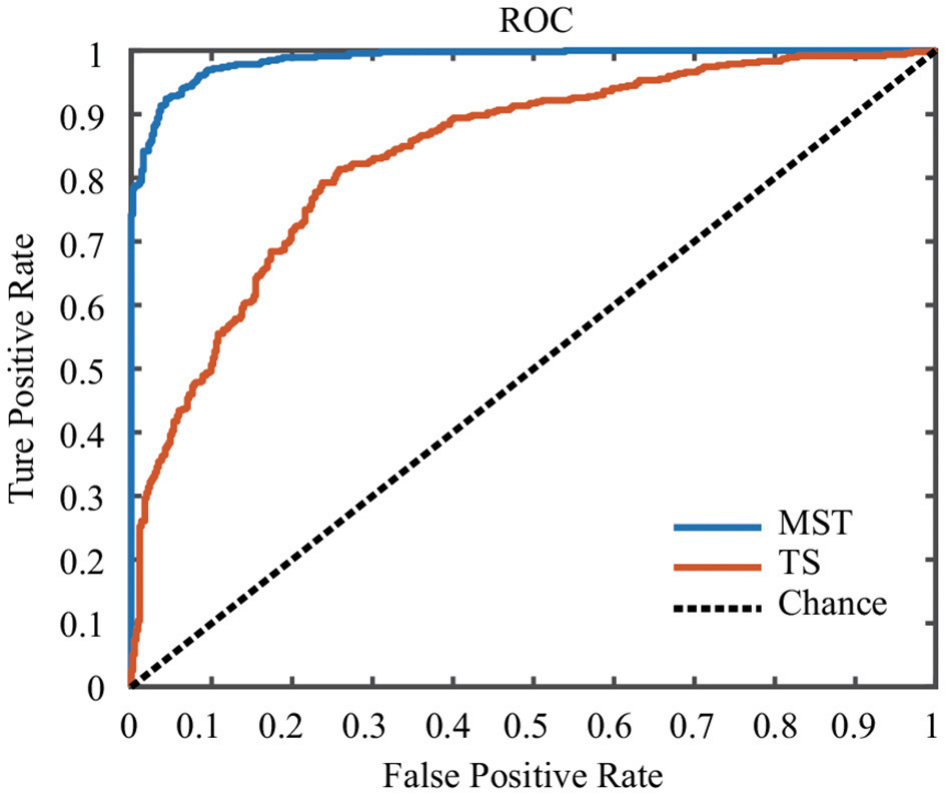
ROC curves of SVM classifiers using MST and TS features.

In this study, the SVM classifier was used to perform the classification. The related measures, including the classification accuracy, sensitivity, specificity and AUC of the ROC curve, were calculated to quantify the results. The MST measures which were significantly different between face and ketch processing were chosen as features to train and test the SVM classifier. Based on the results of above sections, the number of MST features was 82. Figure 6 shows the ROC curves for the SVM classifiers using MST and TS features, respectively. Table 3 presents the classification performance of the MST and TS features. It is obvious that the MST features have better classification performance.

**Table 3:** Classification performance of MST and TS features.

**Feature**	**Accuracy**	**Sensitivity**	**Specificity**	**AUC**
MST	0.9339	0.9437	0.9244	0.9868
TS	0.7566	0.7352	0.7780	0.8375

## 4. Discussion

In this study, the MST topologies of dynamic brain networks of face and ketch processing were compared to explore the mechanism of face processing. The MSTs were constructed based on single-trial EEG data in sequential time windows after stimulus onset over different frequency bands. We found that the MSTs of face processing were more star-like compared with that of ketch processing over theta band in T2 and T3; over alpha band, the MSTs of face processing were more line-like in T1 while more star-like in T2 and T3. The nodes with significant differences in *BC* value and degree were located mainly in the left frontal area, and bilateral ventral visual pathway of the brain in T2 over theta band. Over alpha band, these nodes were located mainly in the left frontal, and right occipito-temporal regions in T2 and T3. In addition, the classification results showed that the special MST topologies in specific time windows over specific frequency bands might reflect the potential mechanism of face processing in human brain. The results were discussed in detail in the following sections.

### 4.1. Topological Organization of Brain Networks

#### 4.1.1. Global Topological Differences of MSTs

In this study, we found that the MSTs were more star-like during face processing than that during non-face processing in T2 and T3 over theta and alpha bands. The MST includes the most important connections of the original network and represents the “high-way” in the network (Wu et al., 2006; Braunstein et al., 2007; Stam et al., 2014; van Dellen et al., 2018). In star-like MST, the topological organization follows a hierarchical pattern (large *Th*) composed of a few layers and some densely connected nodes with most of the nodes serving as periphery within the network (large *L*_*f*_) (Stam et al., 2014; Tewarie et al., 2015). In addition, the star-like MST has smaller diameter that corresponds to shorter path length in original network, which facilitates the communication between regions with long spatial distance and promotes the processing speed. This topological structure of MST contributes to fast and efficient information transfer in the network (Boersma et al., 2013; Cao et al., 2020). Our findings indicated that information transfer and processing were faster and more efficient among brain regions during face processing compared with non-face processing in T2 and T3 over theta and alpha bands. The efficient MST structure might be correspond to the results of previous studies. It is known that the top-down control mechanism plays an important role in face processing (Duchaine and Yovel, 2015; Fan et al., 2020). The top-down processing is associated with neuronal coupling between the frontal and posterior brain regions over theta/alpha bands (Anderson, 2011; Maurer et al., 2015; Bossi et al., 2020; Yin et al., 2020). The top-down processing during face processing is also associated with the alpha band neuronal coupling between parietal and temporal regions (Klimesch et al., 2011; Heyselaar et al., 2018). The top-down mechanism shortens the functional distance between regions with long spatial distance and makes large-scale integration in the brain network. We put forward that the top-down control mechanism might be one of the reasons for the formation of the more star-like topology of brain networks during face processing. Moreover, the duration in which the top-down processing worked during face processing was not clear in previous studies. Based on the above analysis, the findings that the more star-like topology occurred in T2 and T3 suggested that the top-down processing might occur during 100–300 ms after the face onset.

Larger *MaxBC* and *MaxK* were found during face processing compared with that during ketch processing in T2 over theta band, and in T2 and T3 over alpha band. If a network has larger *MaxBC* and *MaxK*, then most of the nodes are connected by several hub nodes, making the network more star-like (Stam et al., 2014; Tóth et al., 2017; Cao et al., 2020). The hub nodes carry the most amount of information transfer and make the processing of information in the network more efficient. Our results indicated that there might exist some hub nodes in the brain network which played an important role during face processing, which was consistent with the results of pervious face studies. In previous studies, brain areas that were very special to face processing were found (Haxby et al., 2000; Ishai et al., 2005; Duchaine and Yovel, 2015; Uono et al., 2017; Fan et al., 2020). In these facial areas, neural activities were distinctive between face and non-face processing. Unlike previous studies, our results based on *MaxBC* and *MaxK* did not provide detailed information about the nodes, nor do they provide information about which nodes were more important during face processing. In this study, the result that larger *MaxBC* and *MaxK* appeared in T2 not T3 over theta band reminded us of N170. N170 that has been considered to be a biomarker of face processing occurs in the duration of 150–200 ms after stimuli onset over the occipito-temporal regions (part of facial areas) (Bentin and Deouell, 2000; Yang et al., 2015). The similar occurrence interval indicated that our result might be related to N170 occurrence during face processing. Moreover, the results that larger *MaxBC* and *MaxK* for face processing in T2 and T3 over alpha band might be consistent with the inter-trail phase coherence (ITPC) study of face processing (Gu et al., 2010). The ITPC study showed that higher alpha band phase synchronization appeared in the duration of 100–270 ms over occipito-temporal regions after stimuli onset during face stimuli compared with non-face stimuli.

The hub nodes facilitate the information integrating in the network, but if they are overloaded, the network will be broken and its efficiency will be seriously reduced (Stam et al., 2014; Yu et al., 2017). Tree hierarchy (*Th*) characterizes the balance between integration and node load in the network. The larger the *Th* value, the more efficient the information integrating of the network, however, the higher the risk of the hub nodes being in an overloaded state. We found that *Th* was larger only in T2 over theta and alpha bands, which indicated that the T2 was important for face processing. In T2, the brain network of face processing organized into a structure with several hub nodes that bore more information processing. According to previous studies (Bentin and Deouell, 2000; Schiltz and Rossion, 2006; Uono et al., 2017), we inferred that this structure of brain network might facilitate the unique processing of face including holistic encoding and configural processing which occurs in 160–210 ms after faces onset. Moreover, it is supposed that overloaded nodes in the brain network may process the most urgent information, but they must not work in an overloaded state for long time to avoid making themselves damaged (Olde Dubbelink et al., 2014; Stam et al., 2014; Fraga González et al., 2016). The result that *Th* was not larger in T3 during face processing over theta and alpha bands indicated that the high loaded nodes restored to normal state after processing of main facial features, although the topology of MST was still more efficient (more star-like MST) for face processing.

We found that MST *PLI* was larger during face processing compared with that during ketch processing in T2 and T3 over theta and alpha bands. MST *PLI* measures the strength of functional connection of the brain on average. Our findings indicated that face processing resulted in enhanced functional connection in the brain network, which were consistent with pervious face processing studies. Specifically, previous EEG studies presented that the functional connection increased between the occipito-temporal regions and other brain regions over theta band, and between frontal/parietal regions and posterior region over alpha band during face processing compared with non-face processing (Yang et al., 2015); moreover, the average functional connection of the brain network increased over theta band during face processing (Yin et al., 2020). Different from these studies in which the temporal information was lost, our results provided the duration in which the functional connection became stronger during face processing.

Besides showing more star-like topology in T2 and T3, we found that the MST of brain network was more line-like during face processing in T1 over alpha band. More line-like MST indicated that the structure of the brain networks tended to be regular (Boersma et al., 2013; Stam et al., 2014; Tóth et al., 2017), which had more clustered regions that contributed to local processing. In this type of network, most of the information processing took place in the clustered regions, with little information transfer between them. Our finding suggested that more clustered regions were formed in the initial stage of face processing and contributed to the processing of basic face features. In previous study, the role of functional brain network's topology in cognition has been postulated by the global workspace theory (GWT) (Baars et al., 2013). GWT proposes that in human cognition, local processing within specific modules occurs in the beginning, after which the local information needs to be integrated within a global workspace that can be identified by a network comprising hub-nodes and inter-modular connections. In our study, dynamical changes of the MST topology from more line-like to more star-like supported the GWT, and indicated that the information separation and integration might be more significant during face processing.

In addition, although the topological organization of MST was more star-like during face processing in T2 and T3 over theta and alpha bands, it could be observed that the difference between face and ketch processing became not significant in terms of several measures in T3. Specifically, the *MaxBC, MaxK*, and *Th* over theta band, and *Th* over alpha band became not significantly different in T3. The results indicated that the brain networks of face and ketch processing were organized dynamically, and their structures trended to be similar after the completion of face feature processing. In previous studies (Bentin and Deouell, 2000; Yang et al., 2015; Uono et al., 2017), they presented that the basic visual features were extracted in the early stage of visual object processing and the unique processing of face occurred during 80–280 ms after stimuli onset, which demonstrated the dynamic nature of face processing. Our results validated this unique property of face processing from the perspective of dynamic brain networks.

#### 4.1.2. Node Feature Differences of MSTs

The node *BC* and degree provide information about the single nodes, reflecting the local processing in the brain network (Sporns et al., 2007; van den Heuvel and Sporns, 2011). The nodes with larger BC and degree regulate the information transfer and processing efficiently in the network. In our study, we found several nodes had significantly larger *BC*, and most of them also had significantly larger degree during face processing. Over theta band, these nodes were mainly located in the left frontal area, and bilateral ventral visual pathway of the brain in T2. This result suggested that neural activity in these nodes might regulate the transfer of face-related information more efficiently and play more important role in face processing. The locations of these nodes were consistent with the previous studies on the “core” and “extent” system of face processing (Ishai et al., 2005; Duchaine and Yovel, 2015), which involved the inferior occipital gyrus, fusiform gyrus, superior temporal sulcus, hippocampus, amygdala, inferior frontal gyrus, and orbitofrontal cortex.

Over alpha band, the nodes with larger *BC* and degree during face processing were mainly located in the left frontal and right occipito-temporal regions in T2, while in the left frontal, and right posterior temporal regions in T3. The results indicated that the mechanism of face processing might be related to the alpha band neuronal synchronization among the left frontal, and right occipito-temporal regions. It has been reported that the alpha band neural activities in left frontal and posterior regions are associated with the top-down processing in face processing (Klimesch et al., 2011; Heyselaar et al., 2018; Yin et al., 2020). However, the time window in which these special neural activities occurs is not clear. Our findings suggested that the T2 and T3 time windows might be the duration in which these neural activities occurred during face processing. Moreover, the special alpha neural activities were found in the right but not in the left occipito-temporal regions. This result might be related to the right hemisphere effect of face processing (Bentin and Deouell, 2000; Duchaine and Yovel, 2015).

In summary, in the previous research on face processing, the functional connectivity and roles of brain regions were investigated. They presented “core” and “extent” systems of face processing. The functional connectivity between the regions in these systems were enhanced, and the regions played specific roles. However, none of them investigated the topology of the brain network during face processing by using graphic theory. Our findings about nodes and MST *PLI* were consistent with the previous studies. Furthermore, as the above analysis, other MST measures provided new perspective on understanding the mechanism of face processing in the brain.

### 4.2. Classification Performance

As discussed above, the dynamical changes of the brain network structures reflected the mechanism of face processing. Our results indicated that the structure of brain network might contain common patterns that could distinguish between face and non-face (ketch) processing based on single-trial EEG data. The results of the machine learning method demonstrated that SVM classifier based on MST measures had better performance compared with that based on conventional temporal features. In this study, we chose MST measures that presented significant difference between face and ketch processing as the features. These features included not only the temporal but also the spatial information of neuronal activities, which characterized the face processing more comprehensively.

Compared with previous related studies, the data analysis method of this study. First, the brain networks were constructed based on single-trial EEG data. In most of previous studies, the EEG segments were firstly averaged over single subject, and then the brain networks were constructed using the averaged EEG data for each subject (Yu et al., 2016; Mehraram et al., 2019; Cao et al., 2020). However, not all the EEG segments were time-locked to human cognitive stages, so the averaging operation might make the intrinsic information broken. The brain networks based on single-trial data could supply many more samples, thus enabling the statistical analysis to find the intrinsic patterns in the data. Second, the SVM classifier was trained using samples from all the subjects. Whereas in previous studies, individual classifiers were constructed for each subject (Wang and Jung, 2011; Barngrover et al., 2016), thus each subject had his own classification performance. But the subject-specific classifiers were hard to apply to other subjects because of the individual differences. In our study, we obtained the common patterns of all the subjects. It can be easily applied to distinguish the brain states regardless of the individual differences.

Finally, we should clarify the data reference issue. When preprocessing the EEG data, data reference should be considered. There are several reference types in EEG study, including the nose, electrode Cz, REST (Dong et al., 2017), and common average reference. In this study, we referenced our EEG data by using common average reference for two reasons. First, in the most of previous studies on face processing, the common average reference was used. In order to compare our result with the results in previous studies (Bentin et al., 1996; Kang et al., 2015; Yang et al., 2015; Foley et al., 2018; Ambrus et al., 2019; Mehraram et al., 2019), we chose the common average reference. Second, Yao's study (Yao, 2001) mentioned that the use of scalp potentials to determine the neural electrical activities or their equivalent sources does not depend on the reference, so we used the common average reference in this study.

### 4.3. Limitations and Future Work

There were several limitations should be paid attention to when interpreting the findings in this study. First, the MST discards the weak connections and only includes the strong connections of the original brain network. It should be noted that, no matter strong or weak it is, each connection may play a certain role in the network. So discarding weak connections may loss some information of the brain network. However, in this study, the advantages of using MST outweighed the disadvantages. For one thing, previous studies have presented that larger neural activities and functional connections occurred during face processing (Yang et al., 2015; Yin et al., 2020). These findings suggested that the selection of strong connections might be rational to study the mechanism of face processing. For another, in contrast to the original brain network-based analysis, the MST-based analysis is not biased by the network size, average degree, or density effects and allows for unbiased comparison between networks of equal size (Stam et al., 2014; Tewarie et al., 2015; van Dellen et al., 2018).

Second, the MST does not supply directional information in the brain network. However, in the brain, the information flow is directional during visual objects processing. Specifically, the basic features of visual objects are extracted firstly on the primary visual cortex, and then the combination and other processings are done along the ventral and dorsal visual pathway or modulated by other functional cortices. In the future study, the methods which can characterize the direction of information flow in the brain network should be explored.

Third, unlike previous studies in EEG segment division, in our study, five EEG segments were selected after the onset of stimulus presentation, each with a duration of 100 ms without overlap. Our data segment was longer than that in previous studies (Bola and Sabel, 2015; Yang et al., 2015; Rizkallah et al., 2018). Longer data segment may lead to severely average effect that reduces the temporal resolution. However, data segments that are too short will lead to excessive computational load. The P1-N170-P2 effect of face processing indicates that the unique processing of face may occur in 0–100, 100–200, and 200–300 ms sequentially (Gu et al., 2010; Yang et al., 2015). Therefore, in order to balance the temporal resolution and computational load, we chose 100 ms as the unit of analysis. Whether 100 ms is the optimal duration should be explored in the future work.

Fourth, recently, the spatial pattern of the brain networks has been investigated to explore the neural mechanism of decision-making and schizophrenia (Li et al., 2019; Si et al., 2020), which is a new perspective to characterize the brain. In our study, we focused on the topological difference of the brain networks during face and non-face processing in different time window, so the spatial pattern of the brain network was not considered. In our future work, we will investigate the spatial pattern of the brain networks during face and non-face processing.

## 5. Conclusions

In this study, we investigated the mechanism of face processing from the spatiotemporal perceptive by using the dynamic brain network method. The MSTs were extracted from the original brain networks and their structures were compared between face and non-face (ketch) processing. The results demonstrated that the MST topology was more line-like in T1 over alpha band, while was more star-like in T2 and T3 over theta and alpha bands. From the graph theory perspective, the special dynamic organization of the brain network facilitated the information transfer and processing during face processing. The locations of the nodes with larger *BC* values and degrees were consistent with the previous studies. The classification performance based on MST measures was superior to that using the EEG time segment. Our study indicated that the special dynamic organization of the brain network might reflect the potential mechanism of face processing in human brain.

## Data Availability Statement

The data and code that support the findings of this study are available from the corresponding author upon reasonable request.

## Ethics Statement

The studies involving human participants as been approved by Ethics Committee of Xidian University and complied with Helsinki Declaration of 1975, as revised in 2000. The participants provided their written informed consent to participate in this study.

## Author Contributions

Data collection, analysis, and writing of manuscript was mainly performed by ZY. YW and SR assisted analysis and writing of manuscript. Study design and supervision was conducted by MD, KY, and JL. All authors contributed to the article and approved the submitted version.

## Conflict of Interest

The authors declare that the research was conducted in the absence of any commercial or financial relationships that could be construed as a potential conflict of interest.
