# Supplementary material for: Special Patterns of Dynamic Brain Networks Discriminate Between Face and Non-face Processing: A Single-Trial EEG Study
Source: Front Neurosci. 2021 Jun 9;15:652920. doi: 10.3389/fnins.2021.652920 (PMC8221185; doi:10.3389/fnins.2021.652920)
Supplement: Supplementary file 1 [file Data_Sheet_1.PDF]

Supplementary Material

1 EEG TIME SEGMENTS FOR CLASSIFICATION

In this study, to test the utility and ability of the MST measures in distinguishing between face and non-face processing, we compared the classification performance of SVM classier trained by MST measures and temporal segment (TS) features set respectively. We chose EEG data of several specific channels as temporal segment feature set. According previous studies (Bentin and Deouell, 2000; Uono et al., 2017; Yang et al., 2015), 11 channels, including TP9, TP7, P7, PO7, O1, Oz, O2, PO8, P8, TP8, and TP10, were chosen, which were red-colored in Figure S1. For each trial, the EEG segments from 50 to 300 ms after the stimulus onset over theta and alpha bands were selected. As the sample rate was 1000Hz, there were 250 points in each EEG segment (one sample). The total number of the TS features was 5500 (250x11x2) of each sample, which was far larger than that of MST features. The number of sample was 7665 (trials) for face and ketch respectively. The cross-validation with five-fold and five repetitions was used to train and test to the classifier. Specifically, there was 6132 samples for training the SVM classifier, and 1533 samples for testing the classifier in each fold.

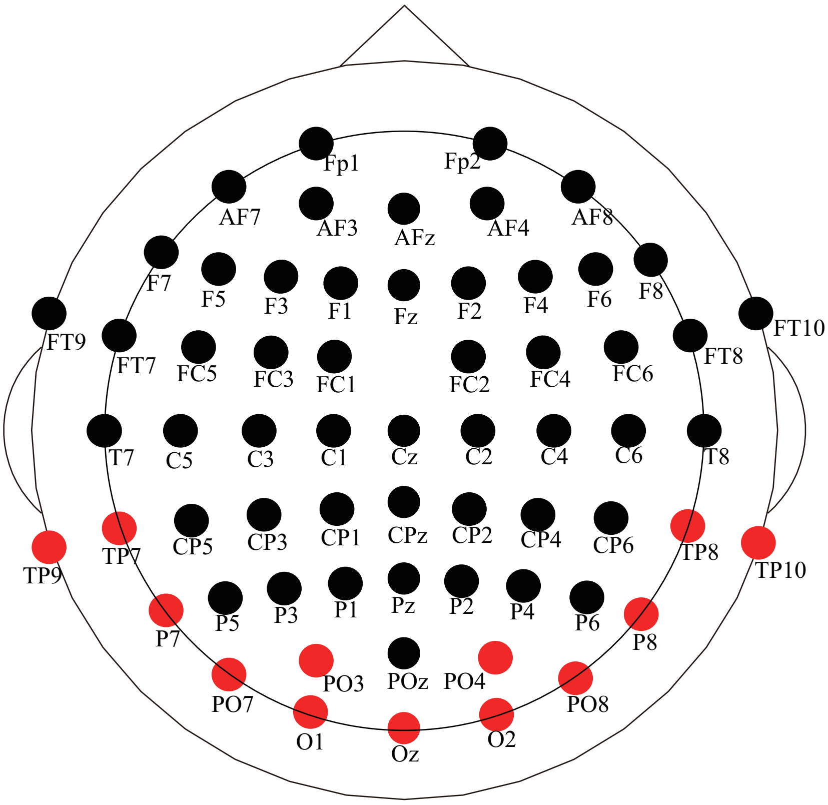

Figure S1. Enter the caption for your figure here. Repeat as necessary for each of your figures

REFERENCES

Bentin, S. and Deouell, L. Y. (2000). Structural encoding and identification in face processing: ERP evidence for separate mechanisms. *Cognitive Neuropsychology* 17, 35–55. doi:10.1080/

026432900380472

Uono, S., Sato, W., Kochiyama, T., Kubota, Y., Sawada, R., Yoshimura, S., et al. (2017). Time course of gamma-band oscillation associated with face processing in the inferior occipital gyrus and fusiform gyrus: A combined fMRI and MEG study. *Human Brain Mapping* 38, 2067–2079. doi:10.1002/hbm.23505

Yang, Y., Qiu, Y., and Schouten, A. C. (2015). Dynamic Functional Brain Connectivity for Face Perception. *Frontiers in Human Neuroscience* 9, 12. doi:10.3389/fnhum.2015.00662
